# Plant-derived polyphenolic compounds for managing schizophrenia: mechanisms and therapeutic potential

**DOI:** 10.3389/fphar.2025.1605027

**Published:** 2025-06-19

**Authors:** Xiaolin Ji, Jianbo Chai, Sihan Zhao, Yonghou Zhao

**Affiliations:** ^1^ The Second Clinical Medical College, Heilongjiang University of Chinese Medicine, Harbin, China; ^2^ Psychiatry Department, Heilongjiang Mental Hospital, Harbin, China

**Keywords:** schizophrenia, plant-derived polyphenols, bioactivity, potential mechanisms, extraction techniques

## Abstract

Schizophrenia is recognized as a severe mental disorder that is marked by psychotic symptoms, which frequently result in profound social stigma and severely hinder occupational functioning. The current research explores the biological activities of plant-derived polyphenolic compounds, specifically highlighting curcumin and epigallocatechin gallate (EGCG), along with other plant polyphenols. Importantly, both curcumin and EGCG demonstrate neuroprotective properties *via* various mechanisms, such as antioxidant and anti-inflammatory effects, neurotransmitter modulation, improvement of mitochondrial function, and stimulation of neurogenesis. These mechanisms play a role in reducing the pathological symptoms of schizophrenia and enhancing cognitive abilities, ultimately improving the overall quality of life for patients. Considering the difficulties linked to existing pharmacological therapies, which frequently present constraints and unwanted adverse effects, plant-derived polyphenolic compounds have attracted interest as potential therapeutic alternatives. These plant compounds hold the potential not only to alleviate the symptoms of schizophrenia but also to enhance cognitive function. They may achieve this through various mechanisms, such as boosting antioxidant defenses, reducing neuroinflammation, balancing neurotransmitters, increasing brain-derived neurotrophic factor (BDNF) levels, and enhancing mitochondrial function. Numerous studies involving animals have shown that polyphenols sourced from plants can markedly increase the levels of brain-derived neurotrophic factor (BDNF), leading to improvements in neurodevelopmental disorders. These compounds also help restore mitochondrial function by replenishing ATP levels and boosting systemic antioxidant capacity through the reduction of serum malondialdehyde (MDA) levels, while simultaneously enhancing the activity of antioxidant enzymes, including superoxide dismutase (SOD). Additionally, they have been reported to lower inflammatory markers, particularly interleukin-6 (IL-6). Moreover, polyphenols play a significant role in regulating neurotransmitter levels by decreasing the concentrations of dopamine and glutamate. Moreover, ongoing research into the structure, classification, and characteristics of polyphenolic compounds, alongside advancements in nanotechnology and metabolic regulation, has opened up innovative delivery strategies for these compounds. Such developments present new opportunities for creating effective and low-toxicity natural derivatives. Future studies are anticipated to support a transition from conventional “single-target” approaches to more comprehensive “multi-target network regulation” therapeutic strategies.

## 1 Introduction

Schizophrenia is a multifaceted psychiatric disorder marked by significant clinical variability, which can be broadly divided into three main categories: positive symptoms, negative symptoms, and disorganized symptoms. Negative symptoms mainly relate to shortcomings in behavior and emotional expression, including diminished affect, lack of motivation, and social withdrawal. In contrast, positive symptoms pertain to irregularities in perception and thought, featuring hallucinations (such as visual or auditory) and delusions (like grandiose ideas). Disorganized symptoms are defined by disturbances in thought processes and emotional expression, which include thought disorder and inappropriate emotional responses. The interaction of these symptom categories adversely affects patients’social interactions and work capabilities, ultimately diminishing their overall quality of life (Nestler et al., 2016; Liddle, 2019). Patients with schizophrenia, whether presenting negative or positive symptoms, exhibit pronounced impairments in attention, information processing, executive functioning, memory, and language abilities. These deficits substantially hinder their capacity to meet the demands of daily occupational tasks (Priyamvada et al., 2021). Positive manifestations of schizophrenia, like hallucinations and delusions, frequently lead to actions that stray from accepted social practices, resulting in public confusion and rejection. In contrast, individuals exhibiting negative symptoms, such as diminished emotional expression, social disengagement, or heightened social anxiety, face challenges in sustaining typical interpersonal connections, which further diminishes their social support. Additionally, negative societal perceptions surrounding the condition, together with the stigma internalized by patients, form a harmful cycle that sustains both social discrimination and isolation (Gerlinger et al., 2013). Schizophrenia is regarded as a highly severe mental disorder, distinguished by the occurrence of psychotic manifestations. The clinical features of schizophrenia are typically classified into three primary categories: negative symptoms (including diminished emotional expression and reduced drive), positive symptoms (such as false beliefs and perceptual disturbances), and cognitive impairments. These manifestations frequently contribute to social stigma and considerable difficulties in work-related functioning (Prestwood et al., 2021). Various neurotransmitters, including dopamine, glutamate, γ-aminobutyric acid (GABA), serotonin, and norepinephrine, play a crucial role in both the onset and advancement of schizophrenia. These chemicals influence not only how signals are transmitted in the brain but are also closely associated with the emotions, thought processes, and behaviors of individuals affected by the disorder (Prestwood et al., 2021). Factors related to prenatal, perinatal, nutrition, and environment play a crucial role in the development of schizophrenia. This includes complications during childbirth, infections in the mother while pregnant, substance misuse, and adverse childhood experiences (Jauhar et al., 2022). While it has not been proven that schizophrenia is transmitted solely through maternal inheritance, some studies suggest that the rate of maternal transmission may be higher (Goldstein et al., 1990; Li et al., 2007). Schizophrenia impacts about 1%–1.5% of the worldwide population, which translates to nearly 1 in every 100 people. The occurrence is slightly more common in males compared to females, with symptoms generally beginning in early adulthood. For males, the occurrence reaches its highest point around the age of 20, while for females, this peak is more subtle and decreases at a slower rate. Interestingly, following the age of 40, the rate of new cases among females surpasses that of males (Howes and Murray, 2014) (Kirkbride et al., 2012).

Second-generation antipsychotic medications (SGAs) serve as a fundamental therapeutic approach for schizophrenia, yet they present notable clinical drawbacks stemming from their metabolic and cardiovascular adverse effects. Research has demonstrated that the usage of these medications can lead to metabolic syndrome and elevate the likelihood of developing cardiovascular conditions (Vasileva et al., 2022). Epidemiological data indicate a concerning link between the prolonged use of both typical and atypical antipsychotic medications and the increased likelihood of developing degenerative Parkinson’s disease (DP). This association is not only significant but also appears to be dose-dependent, meaning that higher doses of these medications may further heighten the risk. Importantly, this risk does not diminish even after patients discontinue the use of the drugs, suggesting a lasting effect on their neurological health. Moreover, the challenges of long-term treatment with antipsychotics are exacerbated by issues such as drug dependence, where patients may find it difficult to stop taking these medications due to withdrawal symptoms, and rapid tolerance, which leads to patients requiring higher doses to achieve the same therapeutic effects. These complications underline the necessity for careful management of antipsychotic treatments. Furthermore, findings from epidemiological studies suggest that both the duration of antipsychotic drug use and the decision to stop treatment after an extended period can substantially increase the risk of developing degenerative Parkinson’s disease (DP). This emphasizes the critical need for ongoing assessment and monitoring of patients on these medications to mitigate potential adverse outcomes (d'Errico et al., 2022). The formation of drug dependence and rapid tolerance exacerbates the complexity of long-term treatment strategies. Against this backdrop, intervention strategies using plant-derived polyphenols have shown promising potential. These natural compounds, acting through multi-target mechanisms—such as neurotransmitter system modulation, anti-inflammatory pathways, and oxidative stress regulation—offer a novel and alternative approach to schizophrenia treatment. Plant-derived polyphenols, sourced from natural sources, provide a cost-effective, accessible, and high-value pharmacological resource. Herbal therapies have long been recognized for their substantial contributions to the management of a diverse range of health conditions. These natural remedies have been used across different cultures and eras, highlighting their enduring significance in traditional medicine. In light of the often uncertain effectiveness of modern pharmacological treatments available for schizophrenia, as well as concerns regarding their potential adverse health effects, there is an increasing trend towards exploring herbal therapies (Datta et al., 2021). This approach provides new avenues for redefining schizophrenia treatment paradigms. Their unique pharmacokinetic properties and low-toxicity advantages stand in stark contrast to conventional antipsychotic drugs. Importantly, studies in ethnopharmacology are increasingly clarifying the scientific underpinnings of conventional herbal remedies used to treat mental health issues. Plant-derived polyphenols exhibit a wide range of biological activities that make them attractive candidates for therapeutic interventions. Among their notable properties are anti-inflammatory effects, which can help to alleviate symptoms associated with various psychiatric disorders. Additionally, these compounds demonstrate significant antioxidant capabilities, which are crucial for protecting neural tissues from oxidative stress. Furthermore, plant-derived polyphenols have been shown to influence neurotransmission processes, potentially enhancing synaptic functions and improving mental health outcomes. Cellular signaling pathways, crucial for ensuring effective communication and function within cells, are also influenced by them. Collectively, these characteristics highlight the potential of polyphenols as novel and effective alternatives to traditional antipsychotic medications, offering hope for innovative treatment strategies in the field of psychiatry (Saleem et al., 2022). Current research focuses on: (1) identifying specific plant-derived bioactive compounds with clinical translation potential; (2) elucidating their mechanisms of action on core pathological features of schizophrenia, such as glutamate dysfunction and dopamine signaling dysregulation; and (3) establishing standardized evaluation systems to ensure their long-term safety and efficacy.

A category of highly significant antioxidant substances known as polyphenols can be found extensively in various plants (Ozdal et al., 2016). Studies have shown that the content of polyphenols varies significantly in different parts of plants, such as the polyphenol content in the pericarp of longan being higher than in the pulp, which may be related to the plant’s living environment (Ke et al., 2023). The antioxidant properties of catechins are primarily attributed to the hydroxyl groups within their catechol moiety, which possess the capacity to form hydrogen bonds with the two oxygen atoms of lipid peroxide radicals (Tejero et al., 2007). The antioxidant activity of flavonoids isolated from celery leaves is determined by the position and number of hydroxyl (-OH) groups on the B-ring of their molecular structure (Wen et al., 2022). Polyphenols belong to a category of organic compounds that are defined by having several hydroxyl (OH) functional groups. They usually present as yellow to brown powdered solids, dissolving in polar solvents like water and ethanol, while remaining insoluble in non-polar solvents such as petroleum ether. Because of their diverse biological activities, including antioxidant, antibacterial, and anticancer effects, polyphenols hold significant potential for application across various domains, including food and pharmaceuticals (Wan et al., 2021). Countries in the West advocate for incorporating polyphenols into the diet, mainly because of their believed essential function in safeguarding long-term health, especially in lowering the likelihood of chronic and degenerative illnesses (Ganesan and Xu, 2017). Research in epidemiology consistently reveals that natural polyphenols hold considerable promise for preventing diseases associated with aging. These advantageous compounds act as scavengers for free radicals and reactive oxygen species (ROS), which often build up due to oxidative stress. In these situations, the body’s intrinsic cellular antioxidants, such as glutathione (GSH), glutathione peroxidase, and superoxide dismutase, frequently fall short in adequately counteracting the overproduction of these detrimental species (Quideau et al., 2011). The presence of multiple hydroxyl groups in polyphenols is mainly responsible for their antioxidant characteristics, along with their capacity to interact with free radicals and bind to metals. When polyphenols react with free radicals, they generate phenolic radicals that do not harm cells (Leopoldini et al., 2011). Additionally, the metal-chelating ability of polyphenolic compounds plays a significant role in cellular function (Wang et al., 2018). Polyphenols are primarily obtained from a variety of plant-based foods, which encompass a wide range of options that include fruits, vegetables, nuts, whole grains, tea, red wine, and chocolate. Notably, certain foods stand out for their high polyphenol content; for instance, berries such as blueberries is particularly rich in these compounds. In addition to berries, green tea, dark chocolate, and red wine are recognized as exceptional sources of polyphenols, providing significant dietary benefits. Moreover, it is worth mentioning that some natural medicines also contain a substantial amount of polyphenols; for example, extracts derived from ginkgo biloba leaves and hawthorn are known to be abundant in these beneficial compounds. (Wan et al., 2021).

## 2 Polyphenols of plant origin

### 2.1 Quercetin

**FIGURE 1:**
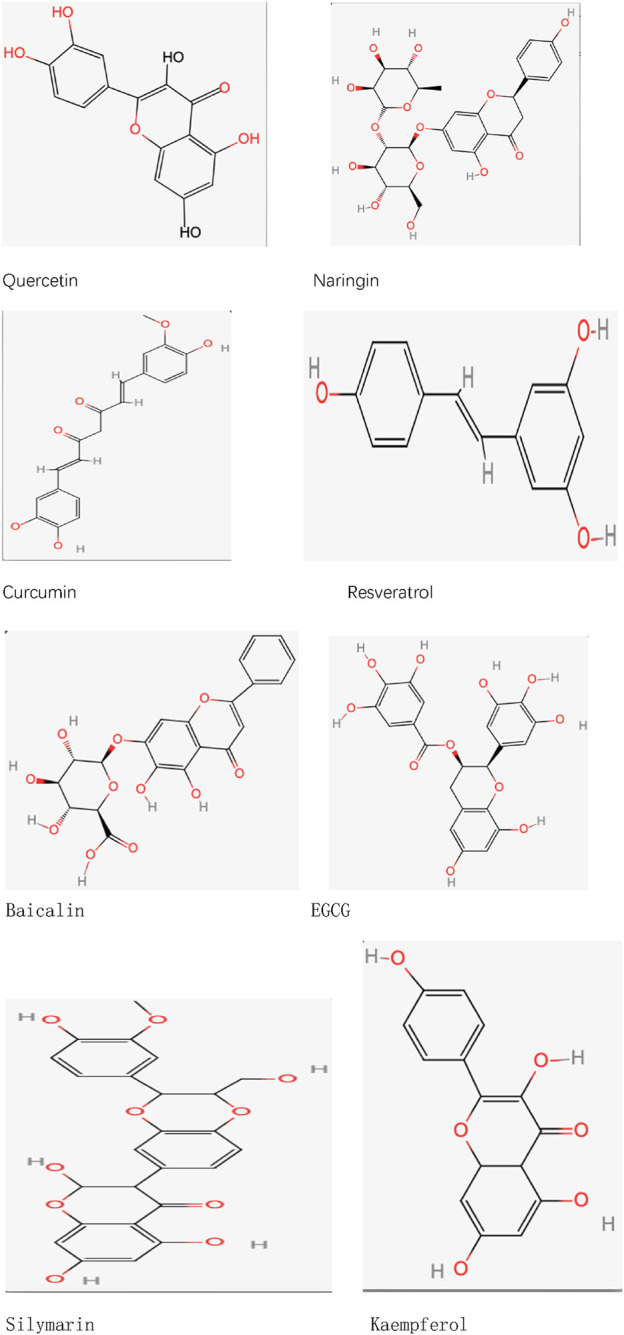
Chemical formula.

Quercetin (C_15_H_10_O_7_), an organic compound within the flavonoid class, is a naturally occurring substance in human nutrition, commonly present in numerous vegetables, fruits, and plants. This compound has been utilized in functional foods as a dietary supplement and is involved in the management of different diseases (Vargas and Burd, 2010). At present, several investigations have established that quercetin, when given in suitable amounts, demonstrates various biological roles, such as anti-inflammatory and antioxidant properties (Li et al., 2016). Quercetin is primarily absorbed by intestinal cells in its glycoside form, after which it is hydrolyzed into aglycones in the intestinal lumen, a process that may involve glucose transporters. Its metabolism primarily occurs in the intestine, with additional metabolism taking place in the liver and blood (Zhang et al., 2018; Andres et al., 2018; Kawabata et al., 2015). Quercetin, a crucial compound derived from plants, exhibits low bioavailability, thereby restricting its effectiveness in clinical settings. To overcome this limitation, scientists have employed multiple strategies aimed at increasing its bioavailability. One such approach involves the glycosylation of quercetin, a modification that greatly improves its water solubility and stability (Sun et al., 2020). Additionally, cocrystallization of quercetin with other molecules (e.g., forming cocrystals with nicotinamide) can effectively improve its solubility and, consequently, its bioavailability (Wu et al., 2020). Researchers have also developed various carrier systems, such as nanoparticles, liposomes, and polymer microspheres, which can effectively encapsulate quercetin and control its release rate, thereby enhancing its bioavailability (Attar et al., 2023). Studies have found that nano-quercetin particles not only improve solubility but also increase cellular uptake, thereby enhancing bioavailability (Ma et al., 2021). Protein-based carriers, such as albumin, have demonstrated encouraging outcomes by interacting with quercetin. This interaction safeguards quercetin from metabolic breakdown while enhancing its stability and bioactivity within the body (Yang et al., 2023) (Figure 1) (Table 2).

### 2.2 Naringin

Naringenin-7-O-rutinoside (C_27_H_32_O_14_), which is scientifically referred to as 5,7-dihydroxyflavone-7-O-rutinoside, represents a category of flavanone glycosides frequently discovered in grape and citrus varieties. Typically, the levels of naringenin-7-O-rutinoside in these fruits are associated with their stage of ripeness, exhibiting greater concentrations in fruits that are not fully ripe (Zeng et al., 2019). Numerous studies have indicated that naringenin-7-O-rutinoside can alter the blood concentrations of certain drugs. When co-administered with these drugs, naringenin-7-O-rutinoside influences drug metabolism by interfering with intestinal enzymes and transporter proteins (Sharma et al., 2021). Although naringenin-7-O-rutinoside exhibits moderate water solubility, it is primarily metabolized in the intestine, where gut microbiota decomposes it into its aglycone form, naringenin, which is then absorbed (Alam et al., 2014). Naringenin-7-O-rutinoside has been proven to possess multiple bioactivities, including anti-apoptotic, anti-atherosclerotic, metal-chelating, and antioxidant properties. Additionally, it is believed to enhance drug absorption and metabolism (Yan et al., 2021). Although there are established technologies for obtaining and refining naringenin-7-O-rutinoside from citrus peel byproducts, its limited solubility and unpleasant taste hinder its direct use. Thus, enhancing its solubility and bioavailability is essential. Shell polysaccharides, recognized for their biodegradability as well as antioxidant and antimicrobial properties, can be utilized in conjunction with naringenin-7-O-rutinoside to create complexes of naringenin-7-O-rutinoside with shell polysaccharides. These complexes enhance the solubility of naringenin-7-O-rutinoside, thereby increasing its bioavailability (Ouyang et al., 2022). Customized deep eutectic solvents (DESs) have shown promise in improving the solubility and bioavailability of naringenin-7-O-rutinoside. For example, a DES formulation consisting of choline chloride (ChCl) and glycerol (Gro) in a 1:3 proportion shows the greatest solubility for naringenin-7-O-rutinoside while preserving neutral properties, which makes it appropriate for its use. Pharmacokinetic studies reveal that the oral bioavailability of naringenin-7-O-rutinoside is doubled in this DES system (Dangre et al., 2023). These advancements offer new clinical prospects for enhancing the bioavailability of naringenin-7-O-rutinoside (Figure 1) (Table 2).

### 2.3 Curcumin

Curcumin (C_21_H_20_O_6_), a lipophilic polyphenol, is recognized for its significant antioxidant, anti-inflammatory, and anticancer properties. This substance is primarily derived from the rhizomes of the Indian plant Curcuma longa (Różański et al., 2021; Chang et al., 2021). First, curcumin exhibits low solubility, which limits its bioavailability following oral administration. Second, its absorption rate is also relatively low, potentially due to its large molecular size, which hinders its ability to cross the intestinal barrier and enter systemic circulation. Furthermore, the metabolic processes of curcumin greatly impact its pharmacokinetic profile. Research indicates that the main metabolites produced from curcumin are tetrahydrocurcumin and curcumin glucuronide, potentially affecting its pharmacological properties (Bolger et al., 2022). To enhance curcumin’s bioavailability, researchers have employed various chemical modification strategies. For instance, forming metal complexes with different metal ions has significantly improved curcumin’s solubility and stability, thereby enhancing its bioavailability and strengthening its antioxidant and anti-inflammatory activities (Shakeri et al., 2019). Furthermore, incorporating curcumin into biocompatible polymers to form solid dispersions or nanoparticles has also been proven to effectively improve its absorption rate and bioavailability *in vivo* (Ma et al., 2019). The methods of chemical modification not only improve the effectiveness of curcumin but also open up new avenues for treating different chronic illnesses (Figure 1) (Table 2).

### 2.4 Resveratrol

Resveratrol (C_14_H_12_O_3_) is a naturally occurring polyphenolic compound known for its various health benefits, including its potential to combat cancer (Kapetanovic et al., 2011). This substance is mainly found in grape skins and red wine (Baur and Sinclair, 2006). It exhibits notable antioxidant and anti-inflammatory properties, positioning it as a promising option for addressing various pathological conditions (Berretta et al., 2020). When taken orally, resveratrol is efficiently absorbed through the gastrointestinal system, achieving peak plasma concentration (Cmax) in approximately 30–240 min post-administration (Nunes et al., 2009; Vaz-da-Silva et al., 2008). Research indicates that after the oral dosing of radiolabeled ^14^C-resveratrol, around 53.4%–84.9% of the total radioactivity can be found in urine, suggesting a high level of intestinal absorption efficiency (Walle et al., 2004). However, resveratrol undergoes considerable first-pass metabolism in the liver (and possibly in the intestine) through mechanisms like glucuronidation and sulfation, which could lead to reduced systemic exposure levels (la Porte et al., 2010). To enhance resveratrol’s bioavailability, strategies such as improving its formulation (e.g., encapsulation into microcapsules or nanoparticles) can be employed to increase its stability and bioavailability. Additionally, gut microbiota can assist in the absorption and metabolism of resveratrol, suggesting that increasing gut microbial populations through probiotics may further improve its bioavailability (Prakash et al., 2024). Such approaches could enhance the therapeutic potential of resveratrol by improving its bioavailability. Furthermore, glycosylation reactions can also enhance resveratrol’s bioavailability. The method utilizes the GH10 xylanase variant (rXynSOS-E236G) to promote the synthesis of 3-O-β-D-xylobiosyl resveratrol, which enhances its solubility in water and improves its bioavailability (Pozo-Rodríguez et al., 2022) (Figure 1) (Table 2).

### 2.5 Baicalin

Baicalein (C_21_H_18_O_11_), a significant flavonoid, is present in the roots of Scutellaria baicalensis. Recent studies indicate that it operates as an agent targeting multiple pathways and functions, exhibiting a range of pharmacological characteristics, including hepatoprotective, anticancer, antibacterial, anti-inflammatory, antidepressant, and antioxidant properties (Chung et al., 2015). Gastrointestinal absorption experiments have revealed that the absorption rate of baicalein exhibits segmental dependence (Song et al., 2017). At first, baicalein is taken up in its original form within the upper gastrointestinal tract, and later, its aglycone variant is absorbed in the colon (Lu et al., 2007). Because of its significant rate of protein binding, baicalein enters the plasma quickly and can sustain its concentration within a specific range (Zhang et al., 2013). Studies examining metabolomics in rat urine and blood suggest that the kidneys and liver are essential for the metabolic processing of baicalein (Zhang et al., 2015). Baicalein is mainly excreted in bile as glucuronide conjugates, with a relatively high biliary excretion rate (Xing et al., 2005). The molecular structure of baicalein includes a flavonoid backbone and a glucose molecule, which contributes to its low solubility in water and, consequently, its poor bioavailability. Studies indicate that methods like creating inclusion complexes with cyclodextrins can greatly enhance both the water solubility and stability of baicalein, ultimately increasing its bioavailability (Jakab et al., 2019). Moreover, utilizing delivery systems in nanomedicine like solid lipid nanoparticles and nanoemulsions has demonstrated a significant enhancement in the bioavailability of baicalein. These innovative technologies not only improve the drug’s solubility and biocompatibility but also facilitate its absorption and distribution throughout the body, indicating a promising future for clinical use (Wang et al., 2023) (Figure 1) (Table 2).

### 2.6 EGCG

EGCG (C_22_H_18_O_11_), a catechin monomer derived from tea leaves, exhibits a range of biological activities, including antioxidant, anti-inflammatory, anti-cancer, and neuroprotective effects, among others (Payne et al., 2022). EGCG is created by the esterification of EGC with gallic acid (GA) and features a structure that consists of a dihydrofuran ring, three benzene rings, and several phenolic hydroxyl groups. While it exhibits significant instability in neutral or alkaline conditions, its stability increases in weakly acidic environments (Chen et al., 2001). Despite its diverse pharmacological activities, EGCG has significant limitations in bioavailability, particularly following oral administration, where systemic absorption is insufficient. These challenges include unfavorable pharmacokinetic properties, limited bioavailability, the impact of first-pass metabolism, reduced accumulation in target tissues, and low targeting efficiency. Additionally, during intestinal absorption, factors such as temperature, pH changes, and oxidative stress can influence EGCG uptake (Cai et al., 2018; Dai et al., 2020). To tackle these limitations, various strategies to enhance the bioavailability of EGCG are currently being investigated by researchers. For example, research indicates that esterification can notably improve the absorption efficiency of EGCG in the intestine (Zhang et al., 2023). Moreover, studies have shown that when EGCG is stabilized using protein carriers like whey protein isolate (WPI) and sodium caseinate (NaCas) within solid-oil-water (S/O/W) emulsion systems, can achieve improved bioavailability and intestinal absorption (Korin et al., 2024). Nanoparticles as drug delivery systems have also shown great potential in enhancing EGCG’s bioavailability. Studies indicate that nanoparticle-based methods, including nanoemulsions, encapsulation, and silica-based nanoparticles, can significantly improve EGCG’s bioavailability while reducing its degradation and clearance rates in the body (Mehmood et al., 2022) (Figure 1) (Table 2).

### 2.7 Silymarin

Silymarin (C_25_H_22_O_10_) is a polyphenolic flavonoid extract primarily composed of 70%–80% baicalein aglycones and 20%–30% fatty acids, along with various other polyphenolic compounds (Neha and Singh, 2016). Silymarin along with its aglycones demonstrates considerable antioxidant, anti-inflammatory, and pro-apoptotic characteristics, leading to a diverse array of biological and pharmacological effects. Although silymarin is non-toxic and possesses remarkable hydrophobic traits, its insufficient solubility in water limits its bioavailability (Zhang et al., 2022). He liver primarily metabolizes silymarin, engaging various enzymes in the process, including the cytochrome P450 enzyme system. In the liver, silymarin undergoes phase II metabolic reactions, forming various metabolites that may exhibit distinct biological activities. Studies have shown that silymarin‘s metabolism not only influences its efficacy but may also affect its safety profile. For instance, silymarin may inhibit the activity of certain drug-metabolizing enzymes, leading to increased concentrations of other drugs in the body and potential drug interactions. The excretion of silymarin occurs mainly through bile and urine (Jaffar et al., 2024; Wu et al., 2009). To enhance its bioavailability, researchers have developed novel formulations, such as silymarin -phospholipid complexes, which have shown higher bioavailability in healthy volunteers compared to traditional silymarin tablets (Méndez-S et al., 2019). Furthermore, it has been demonstrated that nanocrystalline formulations (HM40) greatly enhance the solubility and bioavailability of silymarin (Seo et al., 2024). The use of nanoliposomes as carriers not only enhances the solubility of silymarin but also improves its absorption rates by increasing cell membrane permeability (Barani et al., 2021) (Figure 1) (Table 2).

### 2.8 Kaempferol

Kaempferol (C_15_H_10_O_6_) is widely distributed in vegetables such as kale and spinach (Dabeek and Marra, 2019). It exhibits inhibitory effects on microglial activation, anti-inflammatory properties, and mitochondrial membrane stabilization to prevent oxidative damage (Jin et al., 2023). Kaempferol is typically administered orally in high or low glycoside forms. Due to its fat-soluble properties, kaempferol can be absorbed through passive diffusion, facilitated diffusion, and mechanisms of active transport. (Crespy et al., 2003). Kaempferol is metabolized in the liver to produce glucuronide and sulfate conjugates, with additional metabolism taking place in the small intestine through the action of intestinal enzymes (Chen and Chen, 2013). Kaempferol demonstrates therapeutic effects even at low doses, and its metabolism may generate active metabolites with potential therapeutic value (Alam et al., 2020). As a compound found in diet, the effectiveness of kaempferol in therapy relies on its adequate absorption, metabolism, and elimination (Gee and Johnson, 2001). Nevertheless, the bioavailability of kaempferol is minimal, mainly because of its limited solubility in water and swift metabolism within the body, which limits its clinical and nutritional applications (Park and Choi, 2019; Qian et al., 2016). Encapsulating kaempferol in nanoparticles can significantly enhance its water solubility and bioavailability. For instance, kaempferol nanosuspensions prepared using nanotechnology exhibit substantially improved bioavailability compared to untreated kaempferol (Tian et al., 2020). Furthermore, employing lipid-based formulations may enhance the stability of kaempferol, lower its metabolism within the body, and prolong its pharmacological effects (Patel et al., 2024) (Figure 1) (Table 1).

**TABLE 1:** Polyphenol compounds: Solubility properties, bioavailability challenges, and research methodologies.

Polyphenol compound	Solubility characteristics	Bioavailability challenge	References	Method	References
Quercetin	Insoluble	Low water solubility, Enzyme system optimization effect, Structural limitations	Sun et al. (2020)	Eutectic technology (quercetin-nicotinamide eutectic), Carrier delivery strategies (nanoparticles, liposomes, polymer microspheres, protein carriers)	(Wu et al., 2020) (Attar et al., 2023) (Yang et al., 2023)
Naringin	Insoluble	Poor solubility, Molecular structure characteristics	Ouyang et al. (2022)	Physical complex preparation technology (chitosan oligosaccharides), Green solvent technology (DES)	(Ouyang et al., 2022) (Dangre et al., 2023)
Curcumin	Insoluble	Low water solubility, Metabolic instability, Chemical instability, Rapid elimination, Limited penetration of the blood-brain barrier	Bolger et al. (2022)	Chemical modification (metal complexes), Nanoparticles	Shakeri et al. (2019), Ma et al. (2019)
Resveratrol	Insoluble	Poor water solubility, Rapid metabolism	Pozo-Rodríguez et al. (2022)	Nanoparticles, Probiotics, Glycosylation	(la Porte et al., 2010) (Prakash et al., 2024) (Pozo-Rodríguez et al., 2022)
Baicalin	Insoluble	Active Efflux by ABC Transporters, Low water solubility,, Low permeability, Oxidation instability	Jakab et al. (2019)	Cyclodextrin contains (γ-CD), nanotechnology (nanocrystals, SNEDDS), Inhibits exocytic transporters	(Jakab et al., 2019) (Wang et al., 2023)
EGCG	Soluble	Low absorption efficiency, Chemical instability, First-pass metabolism and enzymatic transformation, Intestinal flora decomposition, Efflux pump effect	Cai et al. (2018), Dai et al. (2020)	Nanocarrier delivery systems (protein carriers, carbohydrate-based carriers, lipid-based carriers), Molecular modification, Combined drug delivery strategies	(Zhang et al., 2015) (Zhang et al., 2023) (Korin et al., 2024) (Mehmood et al., 2022)
Silymarin	Insoluble	Low water solubility, Poor chemical stability, Interactions with digestive enzymes or proteins	Zhang et al. (2022)	Nanotechnology (nanocomposites, liposomes, nanocrystalline technology), phospholipid complexes	(Zhang et al., 2022) (Zhang et al., 2023) (Méndez-S et al., 2019) (Seo et al., 2024) (Barani et al., 2021)
Kaempferol	Insoluble	Large particle size, Poor water solubility, Low dissolution rate	Park and Choi (2019), Qian et al. (2016)	Nanotechnology preparation, Lipid preparations	(Tian et al., 2020) (Patel et al., 2024)

## 3 Toxicology

Research on the toxicology of polyphenols has mainly concentrated on their impact on cells and living organisms, especially the possible toxicity caused by elevated doses, which suggests that careful consideration of dosage and administration is essential when utilizing polyphenols (Sinha et al., 2022). Interestingly, polyphenolic antioxidants derived from plants exhibit both pro-oxidant and antioxidant properties, contingent upon factors such as their metal-reducing potential, chelation behavior, pH conditions, and solubility characteristics (Decker, 1997). The cytotoxicity of polyphenols is mainly associated with their metabolites generated in the body. Certain polyphenols are converted into toxic compounds in the body, which may result in cell damage and apoptosis. The shift of polyphenols from acting as antioxidants to becoming pro-oxidants and possible genotoxic agents primarily relies on the degree of oxidative stress they provoke and the condition of the intracellular antioxidant defense system. For instance, at lower concentrations, EGCG causes a slight increase in oxidative stress, which triggers cellular antioxidant defense mechanisms and enhances antioxidant functions. Conversely, as the concentration escalates, there is a significant surge in the production of reactive oxygen species (ROS), which surpasses the cellular antioxidant capacity, leading ultimately to apoptosis or even necrosis (Babich et al., 2011; Hsuuw and Chan, 2007). Research has demonstrated that a high consumption of polyphenols can lead to damage in liver cells and a decline in their functionality. While polyphenols are known for their antioxidant characteristics, their presence at elevated levels can induce oxidative stress and may exhibit pro-oxidative behaviors *in vitro*. This can result in increased intracellular levels of reactive oxygen species (ROS), subsequently leading to cellular harm. Additionally, polyphenols have been found to exhibit genotoxic effects, potentially causing DNA strand breaks or mutations, which can impair normal cell proliferation and function. Throughout the oxidation process, highly reactive quinone compounds are produced by polyphenols, demonstrating their reactivity *via* covalent bonding with nucleophilic side chains. Furthermore, these quinones have the ability to create adducts with DNA, which may result in genetic mutations. Reactive oxygen species (ROS) are crucial in causing DNA strand breaks and oxidative damage to nucleotides. As polyphenols oxidize into quinones, ROS are generated as a byproduct of O_2_ reduction. Therefore, this mechanism is recognized as an essential factor contributing to their genotoxic effects (Andrés et al., 2023). This genotoxicity typically exhibits dose-dependent characteristics, where low doses of polyphenols may offer protective effects, while high doses can lead to cell damage and toxic reactions. The chemical structure of polyphenols significantly influences their bioactivity and toxicity. For example, polyphenols with a higher number of hydroxyl groups may exhibit stronger antioxidant activity but may also increase the risk of toxicity (Quesada-Vázquez et al., 2024). Polyphenols have the ability to bind with metal ions, which consequently lowers the oxidative reactions catalyzed by metals. Nonetheless, when present in high concentrations, polyphenols can interfere with the typical metabolism and functioning of metal ions (Nowak et al., 2022).

## 4 Extraction method of polyphenols

The extraction of polyphenols often utilizes solvent extraction, which is a conventional approach that has seen extensive application in isolating bioactive compounds from botanical sources. In this method, polar solvents like ethanol, methanol, or water are generally used to draw out polyphenolic compounds from plant materials. Although solvent extraction is simple to operate and cost-effective, it has several drawbacks, this includes issues such as low efficiency in extraction, possible degradation, and the reduction of polyphenol content. Furthermore, traditional solvent extraction methods frequently necessitate extended soaking times and high-temperature procedures, escalating both energy consumption and time expenditure (Abbaspour et al., 2024). In recent years, with growing interest in green extraction technologies, scientists have started to investigate more effective and eco-friendly techniques, including ultrasound-assisted extraction (UAE) and microwave-assisted extraction (MAE). These approaches can improve extraction efficiency in less time while minimizing solvent consumption and reducing environmental effects (Chang et al., 2022). Microwave Superheated Water Extraction effectively disrupts plant cell walls, facilitating the release of polyphenols. Studies have shown that ultrasound-assisted extraction not only shortens extraction time but also operates at lower temperatures, reducing thermal degradation of polyphenols (Fernandes et al., 2019). Ultrasonic−microwave-assisted extraction (UMAE) is an innovative method that employs microwave radiation to simultaneously heat the solvent and the plant material, thereby improving the extraction process’s efficiency. In contrast to conventional solvent extraction and ultrasound-assisted extraction methods, MAE can achieve higher concentrations of polyphenols in a shorter duration and with enhanced extraction effectiveness (Vo et al., 2023). Supercritical fluid extraction (SFE) employs supercritical fluids like carbon dioxide in its supercritical state as solvents for the extraction process. This method is highly efficient and environmentally friendly. It permits extraction at reduced temperatures, thus preventing the breakdown of thermally sensitive substances, and facilitates the selective retrieval of desired compounds (Herzyk et al., 2024) (Table 2).

**TABLE 2:** Extraction method of polyphenols.

Method	Efficacy	Cost	Scalability	Eco-friendliness	References
UAME	High extraction efficiency , High retention of biological activity	Low solvent consumption, Energy consumption optimization	Suitable for industry, Flexible parameters	Green solvents, Waste reduction	Vo et al. (2023)
SFE	**High selectivity and efficient extraction**, **High purity of the extract**	High initial investment, Low long-term operating costs	Wide industrial applications	Green solvents, Products can be recycled	Herzyk et al. (2024)
MWE	High extraction efficiency, Selective extraction	High initial investment, Low long-term operating costs	Industrial applications remain to be verified	Green solvents, Recyclable products, Waste reduction	Fernandes et al. (2019)

## 5 Nanotechnology in enhancing polyphenol delivery

Recent progress in nanotechnology illustrates considerable promise in improving the delivery and effectiveness of polyphenolic compounds aimed at treating neurological conditions, including schizophrenia. Significantly, nanocarriers can address the difficulties presented by the blood-brain barrier by enhancing the lipophilicity of therapeutic agents, which aids in their absorption into the brain. In addition, the application of nanotechnology boosts both drug stability and bioavailability; encapsulation methods safeguard the structural integrity of the compounds while extending their effective concentrations in the brain *via* controlled release systems. Crucially, nanocarriers facilitate targeted delivery, guaranteeing accurate placement of therapeutics in areas affected by neuroinflammation (Rajendran et al., 2022).

## 6 Polyphenols improve schizophrenia

Recent findings indicate that these naturally present bioactive compounds effectively manage the concentrations of molecules related to oxidative stress, such as glutathione (GSH), superoxide dismutase (SOD), and catalase (CAT), while also decreasing the amounts of lipid peroxidation products like malondialdehyde (MDA). Additionally, they influence the concentrations of pro-inflammatory cytokines such as tumor necrosis factor-α (TNF-α) and interleukin-6 (IL-6), which contribute to alleviating primary symptoms, including cognitive impairments. These results offer essential theoretical backing for the prospective clinical use of polyphenolic compounds in treating schizophrenia (Ben-Azu et al., 2024a) (Mert et al., 2019) (Farkhakfar et al., 2023) (Table 3).

**TABLE 3:** Polyphenols improve schizophrenia.

Method	Dose	Modle	Target		Medicine
Polyphenols improve schizophrenia	50, 100 mg/kg	Swiss male mice	MPO, TNF-α, IL-6, BDNF, GSH, CAT, SOD	Ben-Azu et al. (2024a)	Silymarin
	25 mg/kg, 50 mg/kg	Balb-C mice	MDA, SOD, GPx	Mert et al. (2019)	Quercetin
	20 mg/kg, 80 mg/kg	male mice	MDA, GPx, CAT, SOD	Farkhakfar et al. (2023)	Resveratrol

## 7 Effects of polyphenols on the pathogenesis of schizophrenia

### 7.1 Regulates neurotransmitters

#### 7.1.1 Lower dopamine

The dopamine hypothesis stands out as a leading theory in the study of schizophrenia, suggesting that the onset of the disorder is closely linked to disruptions in the brain’s dopamine system. Enhanced dopamine signaling has been associated with schizophrenia-like behavioral manifestations (Simpson and Kellendonk, 2017; Grace, 2016). Dopamine receptors can primarily be divided into two categories: the D1-like receptors (which include D1 and D5) and the D2-like receptors (consisting of D2, D3, and D4). Interestingly, D3 receptors exhibit a stronger affinity for dopamine compared to D2 receptors, which in turn possess a higher affinity than D1 receptors. Research conducted post-mortem on patients with schizophrenia has shown an increase in dopamine receptor levels (McCutcheon et al., 2020). Research utilizing neuroimaging techniques suggests that individuals with schizophrenia exhibit heightened presynaptic dopamine activity in the posterior striatum (McCutcheon et al., 2018). The striatum, as the core of dopamine-sensitive basal ganglia circuits, is closely associated with movement disorders and reward deficits linked to schizophrenia. Studies have indicated that increased production and secretion of dopamine in the striatum are associated with a worsening of psychotic symptoms in individuals, a finding supported by neuroimaging studies (McCutcheon et al., 2020; Weinstein et al., 2017). Recent research has offered evidence that aligns with the theory suggesting that schizophrenia is linked to a condition of dopamine deficiency in the cortical region (Slifstein et al., 2015; Rao et al., 2019; Frankle et al., 2022). Reduced cortical dopamine function may be interconnected with the observed increase in striatal dopamine activity in schizophrenia patients. Research in clinical settings indicates that lower levels of cortical dopamine may result in increased dopamine levels in the striatum, a connection that has similarly been noted in people diagnosed with schizophrenia (Frankle et al., 2022; Pycock et al., 1980; Clarke et al., 2014). Dopamine signaling within the cortex plays a crucial role in the proper functioning of attention, working memory, and executive functions. As a result, when dopamine does not operate correctly in schizophrenia, it can impede these cognitive processes (Cools and D'Esposito, 2011; Floresco and Magyar, 2006; Nieoullon, 2002).

Silymarin opposes and prevents the rise of dopamine levels in the striatum, prefrontal cortex, and hippocampus that is induced by ketamine, which in turn enhances excessive motor behavior, stereotypical actions, memory impairments, and social disengagement (Ben-Azu et al., 2024a). In a study utilizing a bilateral olfactory bulbectomy (OBX) model, it was demonstrated that curcumin, the primary component found in turmeric can help mitigate the reduction in dopamine levels seen in the prefrontal cortex. This discovery could offer valuable perspectives on the connection between reduced cortical dopamine levels and heightened striatal dopamine concentrations (Xu et al., 2005). A study conducted on fruit flies found that quercetin lowers the dopamine levels within brain homogenates while elevating its oxidized metabolite, 3,4-dihydroxyphenylacetic acid (DOPAC). These findings indicate that the influence of quercetin on sensitivity and locomotor sensitization may, in part, be attributed to the decrease in dopamine observed in the flies treated with this compound (Filošević Vujnović et al., 2023). Research on resveratrol revealed that, under hypoxic conditions, it decreases dopamine release in rat striatal slices and shows potential in improving pathological changes caused by hypoxia-ischemia, indicating its promise as a therapeutic agent (Gürsoy and Büyükuysal, 2008) (Table 2).

#### 7.1.2 Glutamate reduction

The primary excitatory neurotransmitter in the central nervous system is glutamate, which is widely distributed throughout the brain due to the presence of glutamatergic neurons. Those who exhibit elevated levels of glutamate in the hippocampus have an increased likelihood of developing psychosis (McCutcheon et al., 2020; Bustillo et al., 2019). Impairment of glutamate activity is associated with the underlying mechanisms of schizophrenia. Research indicates that individuals diagnosed with this condition display notably higher absolute levels of glutamate in both the prefrontal cortex and hippocampus, and there is a correlation between elevated glutamate concentrations in the prefrontal region and diminished overall psychosocial functioning (van Elst et al., 2005). The precise physiological processes associated with schizophrenia are not well understood. However, the glutamate hypothesis proposes that reduced functionality of N-methyl-D-aspartate receptors (NMDARs) found on inhibitory γ-aminobutyric acid (GABA) ergic interneurons leads to insufficient activation of neurons that secrete glutamate, resulting in weakened inhibitory signaling. This chain of events ultimately causes an increase in glutamate release, which may lead to the onset of psychotic symptoms (Inta et al., 2010; Harrison and Weinberger, 2005; Li et al., 2024). An increasing amount of research indicates that abnormalities in glutamate neurotransmission could be fundamental to the primary pathological characteristics of schizophrenia. A meta-analysis focused on glutamate concentrations in individuals with schizophrenia showed significantly higher variability in glutamate metabolites among these patients than in healthy controls, especially in brain areas like the medial prefrontal cortex, dorsolateral prefrontal cortex, and thalamus. This variability was apparent in the levels of glutamate, glutamine, and Glx. Moreover, individuals experiencing more severe symptoms generally displayed greater variability in the levels of glutamate metabolites, which supports the notion of elevated glutamate levels in schizophrenia (Merritt et al., 2023). For individuals suffering from treatment-resistant schizophrenia, the levels of Glx and glutamate within the anterior cingulate cortex (ACC) play a crucial role in speech learning capabilities, in addition to aiding in classification tasks and enhancing cognitive functions (Huang et al., 2023; Pillinger et al., 2019).

Quercetin, a natural flavonoid known for its neuroprotective properties, has been demonstrated to hinder the release of glutamate triggered by depolarization in the nerve terminals of rat cerebral cortex. This inhibitory effect is associated with a reduction in voltage-dependent Ca^2+^ influx and the suppression of PKC and PKA activities (Lu et al., 2013). Glutamic acid decarboxylase (GAD), an enzyme that depends on pyridoxal phosphate (PLP) facilitates the irreversible transformation of L-glutamate into γ-aminobutyric acid (GABA). A crucial stage in the synthesis of GABA involves a decrease in GAD activity, which directly affects GABA production and can result in a relative rise in glutamate levels because of the hindered conversion of L-glutamate into GABA. Nevertheless, research indicates that naringenin is capable of reversing and halting the depletion of GAD in the hippocampus, striatum, and prefrontal cortex (Lee et al., 2019; Ben-Azu et al., 2024b). Baicalein stabilizes the glutamine synthetase (GS) protein, preventing its degradation by the 20S proteasome under oxidative stress, thus facilitating the removal of excitatory amino acids like glutamate and mitigating neuronal injury resulting from the buildup of glutamate (Song et al., 2020). Silymarin and curcumin have been found to lower glutamate levels, potentially by increasing BDNF levels. Specifically, curcumin restores TrkB phosphorylation induced by glutamate. However, while silymarin reduces glutamate levels, its effects on BDNF are primarily observed in male mice, with no significant changes in female mice. This implies that silymarin could possess potential therapeutic benefits for cognitive decline in males (Shokouhi et al., 2020; Wang et al., 2008). I A study investigating the impact of resveratrol on C6 glioma cells revealed that resveratrol affected both the uptake of glutamate and the activity of glutamine synthetase (GS). In particular, at concentrations between 0.1 and 100 μM, resveratrol triggered a proportional increase in glutamate uptake, whereas at 10 and 100 μM, there was a notable enhancement in GS activity (dos Santos et al., 2006). This is critical for reducing glutamate levels and maintaining its concentration at low levels. Finally, EGCG effectively blocks glutamate excitotoxicity induced by the glutamate transporter inhibitor threo-3-hydroxyaspartic acid (THA). After THA treatment, glutamate levels in the culture medium significantly increased; however, co-treatment with EGCG reversed this effect (Yu et al., 2010) (Table 2).

### 7.2 Improve neurodevelopmental abnormalities

Brain-Derived Neurotrophic Factor (BDNF), which belongs to the neurotrophin family, is situated on chromosome 11p14.1 in the human genome, specifically positioned on the short arm (p) at 14.1 (Urbina-Varela et al., 2020). BDNF plays a crucial role in multiple aspects of neuronal growth and functioning, including synaptic plasticity, neurotransmitter release, and the differentiation, proliferation, and survival of neurons. Furthermore, BDNF contributes to the development and preservation of dopaminergic, GABAergic, and cholinergic neurons, enhancing effective synaptic transmission and boosting learning, memory, and cognitive abilities. Moreover, it participates in regulating neuroinflammation and exhibits neuroprotective characteristics within the nervous system (Shkundin and Halaris, 2023). BDNF enhances neuroprotection by increasing levels of anti-apoptotic proteins from the Bcl-2 family and caspase inhibitors. It reduces the function of pro-apoptotic proteins such as Bax and Bad, while simultaneously boosting the synthesis of antioxidant enzymes, highlighting its significant antioxidant capabilities. (Marosi and Mattson, 2014). Considering the significance of disrupted neurodevelopment in the onset of schizophrenia (SCZ), BDNF has surfaced as a potential biomarker for the disorder due to its close ties to neurodevelopment (Nieto et al., 2013). Variations in BDNF levels and issues with BDNF signaling pathways have been connected to the development of schizophrenia (Nieto et al., 2021). Moreover, the expression levels of BDNF found in the peripheral blood of patients with schizophrenia are related to the extent of cognitive impairment. This indicates that reduced BDNF levels could worsen the severity of the disease and are strongly linked to cognitive decline (Fernandes et al., 2015). BDNF serves various functions in alleviating the pathological mechanisms associated with schizophrenia by facilitating synaptic restoration, reestablishing neurotransmitter equilibrium, reducing inflammation, and improving both the effectiveness of pharmacological treatments and the response to environmental strategies. The genetic variations, such as rs6265, along with BDNF expression levels, show potential as biomarkers that could inform personalized therapeutic approaches (Shkundin and Halaris, 2023).

Polyphenols, as multi-target modulators, exhibit a broad spectrum of therapeutic effects on schizophrenia by influencing various pathological mechanisms. Silymarin, for instance, demonstrates potent antioxidant properties, mitigates oxidative stress, and modulates multiple neurotransmitter systems. Notably, it reverses the reduction of BDNF levels induced by ketamine in schizophrenia models, highlighting its therapeutic potential (Ben-Azu et al., 2024a). Quercetin activates the BDNF-TrkB-mediated signaling network, which regulates neuronal survival and plasticity. The initiation of the TrkB receptor is a vital aspect of this pathway, which results in the stimulation of PI3K/Akt and the subsequent improvement of BDNF expression and its function (Yao et al., 2012). Similar effects have been observed with kaempferol (Yan et al., 2019). Curcumin modulates the BDNF-TrkB-mediated signaling pathway by regulating the CREB/BDNF axis and also targets the PI3K/Akt pathway to modulate Wnt signaling, thereby elevating BDNF levels (Lou et al., 2024). Resveratrol, as an activator of SIRT1, enhances BDNF transcription through deacetylation, increasing its expression and promoting interactions with the TrkB receptor. In MK-801-induced schizophrenia rat models, resveratrol improves cognitive and motor deficits by upregulating SIRT1 expression (Niu et al., 2020). EGCG increases BDNF expression by activating the ERK/CREB/BDNF pathway and also enhances CREB phosphorylation in the brain, promoting BDNF synthesis and secretion (Mi et al., 2017). Furthermore, baicalein has been shown to alleviate ketamine-induced neurotoxicity in developmental rat models by modulating the CREB/BDNF signaling pathway (Zuo et al., 2016) (Table 2).

### 7.3 Improve oxidative stress

Signs of oxidative stress primarily include lipid peroxides such as malondialdehyde (MDA), oxidized protein products, and indicators of DNA damage due to oxidation. Excessive amounts of reactive oxygen species (ROS) and reactive nitrogen species (RNS) are harmful byproducts produced during aerobic respiration, which cause oxidative damage to lipids, proteins, and nucleic acids (Valko et al., 2007). A hallmark of schizophrenia is the reduction in the levels of redox substrates, particularly glutathione (GSH), in areas of the brain linked to the disorder, including the anterior cingulate cortex, striatum, prefrontal cortex, and thalamus (Reyes-Madrigal et al., 2019; Das et al., 2019; Do et al., 2000). Studies show that people diagnosed with schizophrenia exhibit higher serum malondialdehyde (MDA) levels and reduced quantities of antioxidant enzymes, like superoxide dismutase (SOD), in addition to diminished endogenous antioxidants, including glutathione (GSH). This observation reinforces the idea that their antioxidant capacity is impaired (Buosi et al., 2021). One study found that MDA levels were elevated in patients with negative symptoms (Bošković et al., 2011), while GSH levels were associated with cognitive functioning (Ransohoff and Perry, 2009). Oxidative stress can damage parvalbumin interneurons and oligodendrocytes, contributing to the development of schizophrenia. Parvalbumin interneurons (PV-INs), which are implicated in schizophrenia, play a role in inhibiting GABAergic synaptic networks, helping to maintain the balance of excitation-inhibition and signal coordination between brain regions. A lack of GSH makes parvalbumin-positive interneurons especially susceptible to oxidative harm, which is marked by the breakdown of the perineuronal net and a decline in neuronal population. At the same time, reduced levels of GSH hinder the functionality of NMDAR, amplifying oxidative stress in parvalbumin-positive interneurons. Together, these issues disturb the balance between excitation and inhibition in the brain. (Hu et al., 2014; Perkins et al., 2020).

Quercetin significantly affects glutathione (GSH) and its oxidized form, GSSG, showing its capacity to combat oxidative damage in the brain (Dora et al., 2021). Moreover, quercetin impacts the Keap1/Nrf2/HO-1 signaling pathway by reducing the concentrations of reactive oxygen species (ROS), increasing malondialdehyde (MDA) levels, and enhancing superoxide dismutase (SOD) activity, thereby illustrating its antioxidant capabilities (Cheng et al., 2024). An essential process for addressing oxidative stress involves the stimulation of the signaling pathway related to nuclear factor erythroid 2 (Nrf2). Nrf2 undergoes negative regulation through its interaction with Kelch-like ECH-associated protein 1 (Keap1), resulting in the activation of antioxidant response element (ARE) sequences, which subsequently triggers the expression of genes that provide protective effects (Ma, 2013). Compounds such as EGCG (Kim et al., 2022), curcumin (Liao et al., 2023), naringenin (Garabadu and Agrawal, 2020), baicalein (Wang et al., 2021) can also activate Nrf2-related signaling pathways and elevate antioxidant levels (Rashid et al., 2014) (Khan et al., 2019). The administration of kaempferol mitigates oxidative stress and memory impairments through the modulation of the GSK3β-Nrf2 signaling pathway, which, in turn, lessens neurotoxicity (Hussein et al., 2018) (Table 2).

### 7.4 Improved immune response

Neuroinflammation involves the activation of microglial cells. When injury or disease occurs, these cells are triggered and engage in the activation of cytokines, which play a crucial role in the inflammatory process. When activated, microglial cells have the ability to enhance immune responses within the central nervous system (CNS), resulting in a range of behavioral effects (Fond et al., 2020; Frank et al., 2007). Under normal conditions, although microglial cells are in a resting state, they still influence the brain microenvironment. When the brain encounters injury, local inflammation, or responds to systemic inflammation, microglial cells are activated, exhibiting specific changes such as morphological alterations, enhanced expression of surface markers, promotion of T-cell activity, and secretion of various inflammatory factors (Dantzer, 2004; Perry et al., 2010; Müller, 2018).

Evidence has long supported the established connection between schizophrenia and the immune system, suggesting an association between schizophrenia and both infections and systemic inflammation (Brown and Derkits, 2010; Khandaker et al., 2012; Khandaker et al., 2013; Miller et al., 2011). Cytokine levels and different inflammatory mediators, such as tumor necrosis factor-alpha (TNF-α) and interleukin-6 (IL-6), are elevated in the peripheral blood of people diagnosed with schizophrenia. These increased markers are strongly linked to both the severity of the illness and the expression of psychotic symptoms (Li et al., 2021; Upthegrove and Khandaker, 2020). Within the nervous system, interleukin-6 (IL-6) in the blood binds to receptors on the vagus nerve. Subsequently, signals are transmitted retrogradely through the axons to the brainstem and ultimately reach the nuclei of the hypothalamus. After these signals enter the central nervous system, the effect of cytokines is intensified, resulting in the activation of microglial cells. These activated cells then generate pro-inflammatory cytokines, chemokines, and proteases (Dantzer et al., 2008). These signaling molecules activate indoleamine 2,3-dioxygenase 1 (IDO1), which is an enzyme that plays a crucial role in the metabolism of tryptophan through the kynurenine pathway. This mechanism results in elevated levels of kynurenine and its byproduct, quinolinic acid, both of which play a role in adrenergic neurotransmission (Walker et al., 2013). Kynurenic acid, known for its antagonistic effects on the NMDA receptor, disrupts glutamatergic signaling when present in elevated levels, contributing to dysregulation of various pathways. Simultaneously, inflammatory cytokines can hinder the signaling processes of other neurotransmitters like dopamine, resulting in deficits in cognitive abilities and psychiatric functions. Additionally, kynurenic acid affects the Wnt/β-catenin signaling pathway, worsening neuroinflammatory damage within the brain and ultimately leading to symptoms linked to schizophrenia (Vallée, 2022). This occurrence is related to the cognitive, negative, and positive symptoms connected to schizophrenia and could also lead to difficulties in mood, perception, and cognitive function (Reichenberg et al., 2001).

Recent research indicates that the use of curcumin promotes the production of anti-inflammatory cytokines, specifically IL-4 and IL-10, in BV-2 microglial cells that are subjected to lipopolysaccharide (LPS) exposure. In addition, curcumin increases the levels of suppressor of cytokine signaling (SOCS)-1 while reducing the phosphorylation of JAK2 and STAT3 (Porro et al., 2019). The signaling pathway known as JAK-STAT is made up of Janus kinases (JAK) along with signal transducers and transcriptional activators (STAT), is a highly efficient and specific signaling cascade that transmits extracellular signals to the nucleus with minimal intermediate steps. The basic signaling process involves the activation of JAK and the phosphorylation of STAT upon ligand-receptor binding, resulting in the creation of STAT dimers that migrate into the nucleus to control gene expression. The JAK family of tyrosine kinases, which includes JAK1, JAK2, JAK3, and Tyk2, reacts to a variety of stimuli, including growth factors, interleukins, interferons, and hormones. The STAT family comprises seven members, ranging from STAT1 to STAT7, and is tasked with the regulation of crucial nuclear genes that are important for processes such as apoptosis and cell cycle control, among other biological functions. Furthermore, it is recognized that the JAK-STAT signaling pathway interacts with both the MAPK and PI3K-AKT pathways. Studies suggest that the JAK2-STAT3 pathway plays a critical role in tumor development, inflammatory responses, and oxidative stress (Zhou et al., 2019; Zhang and Yang, 2021). Quercetin, by modulating factors within the JAK signaling pathway, demonstrates possible therapeutic benefits for diseases related to the immune system. Additionally, this mechanism could offer novel perspectives on treating schizophrenia (Muthian and Bright, 2004). Research using the BTBR mouse model indicates that resveratrol significantly decreases the generation of pro-inflammatory cytokines in a living organism and prevents the activation of the JAK1-STAT3 signaling pathway. This discovery indicates that resveratrol possesses strong anti-inflammatory properties and also efficiently modulates the JAK1-STAT3 signaling pathway, providing important theoretical support for future therapies targeting neuroimmune functional disorders (Ahmad et al., 2018). Through its effects on the JAK/STAT3 signaling pathway, baicalein has demonstrated the ability to promote the transformation of microglial cells toward the anti-inflammatory M2 phenotype and significantly enhance the phagocytic capacity of BV-2 cells, which act as a model for microglial cells (Li et al., 2022). It has been demonstrated that IL-6 induces the activation of STAT3. The compound EGCG is capable of obstructing this activation by preventing the signaling pathway of JAK/STAT3 that is mediated by IL-6 (Lin et al., 2012) (Table 2).

### 7.5 Improve mitochondrial energy metabolism

Mitochondria, commonly known as the “powerhouses” of the cell, generate ATP through a process called oxidative phosphorylation, which is vital for supplying energy necessary for proper brain function. In the mitochondrial matrix, the tricarboxylic acid (TCA) cycle promotes the reduction of flavin adenine dinucleotide (FAD) and nicotinamide adenine dinucleotide (NAD^+^), which are critical for numerous cellular functions (Kerr et al., 2017). In individuals with schizophrenia, impairments in brain cell function, synaptic plasticity, and cortical circuitry disruption may stem from energy metabolism damage, processes predominantly regulated by mitochondria (Ni et al., 2024; Fišar, 2023; Sullivan et al., 2018; Duarte and Xin, 2019). The schizophrenia risk gene DISC1 interacts with Mitofilin, a mitochondrial inner membrane protein, thereby influencing mitochondrial function. This discovery supports the notion that mitochondrial dysfunction contributes to energy metabolism imbalances in schizophrenia (Park et al., 2010). Research has additionally shown that in the brain samples taken post-mortem from individuals with schizophrenia, several genes associated with mitochondrial quality control mechanisms are improperly regulated, resulting in decreased expression of genes that play a role in mitochondrial respiration and ATP synthesis (Suárez-Méndez et al., 2020). Reduced activity has specifically been noted in Complex I within the dorsolateral prefrontal cortex, as well as in Complex IV in the prefrontal cortex. Furthermore, both Complexes I and III exhibit decreased activity in the temporal cortex and basal ganglia. In addition, Complexes I, III, and IV in the caudate nucleus, along with Complex IV in the putamen, also demonstrate a decline in activity (Sullivan et al., 2018; Das et al., 2022; Iwata, 2019; Roberts, 2021). The results suggest a reduced capacity for mitochondrial oxidative phosphorylation, which may compromise the brain’s energy provision and could consequently result in cellular dysfunction or death, possibly associated with the pathogenesis of the disorder.

Quercetin has shown the capacity to rejuvenate cellular ATP levels, stabilize the mitochondrial membrane potential, and improve mitochondrial structure, thereby enhancing mitochondrial function. Additionally, research indicates that quercetin notably influences resistance to oxidative stress and inflammation, underscoring its ability to engage various biological pathways (Kang et al., 2020). The flavonoid compound naringenin has been found to improve mitochondrial function and cognitive function in the brains of mice with high-fat diet-induced impairments. Research suggests that naringenin improves the mitochondrial membrane potential (MMP) within the hippocampus, lowers levels of reactive oxygen species (ROS), and boosts ATP levels, thus mitigating mitochondrial injury. This impact may be linked to its stimulation of AMPK (Wang et al., 2015). Similarly, resveratrol can improve mitochondrial function in the hippocampus by addressing issues such as increased peroxide generation, reduced membrane potential, and decreased ATP synthesis, thereby altering depressive-like behaviors (Chen et al., 2017). Baicalein can also activate mitochondrial autophagy in an AMPK-dependent manner, reducing cortical ketone-induced damage to hippocampal neurons. This mechanism is similar to that of naringenin (Liu Y. et al., 2024). A study on human neuronal cell models (NT2-N cells) treated with baicalein revealed that baicalein significantly improved basal mitochondrial respiration, ATP turnover, and maximal mitochondrial capacity, indicating its ability to enhance mitochondrial function (Liu ZSJ. et al., 2024). Research demonstrates that EGCG has the potential to enhance the protective functions of ADSCs located in the cerebral cortex of rats. By activating the SIRT1 pathway, EGCG promotes mitochondrial biogenesis and functionality, increases the activity of cytochrome C oxidase, and improves the mitochondrial membrane potential as well as the efficacy of oxidative phosphorylation in both neurons and astrocytes. As a result, there is a notable increase in ATP synthesis, which contributes to the maintenance of cellular energy resources (Hsieh et al., 2020). As a stimulator of the mitochondrial Ca^2+^ unidirectional transport channel (mCU), cinnamic acid enhances the activity of mCU, leading to an increase in mitochondrial Ca^2+^ absorption. Additionally, cinnamic acid maintains mitochondrial function by improving the flux of pyruvate and tricarboxylic acid (TCA) cycles (Chitturi et al., 2019). Curcumin, a naturally occurring substance known for its various biological functions, has the ability to enhance the production of energy in mitochondria and elevate ATP concentrations. A study in mice demonstrated that curcumin supplementation improved mitochondrial function and increased ATP levels. Furthermore, curcumin can activate mitochondrial signaling pathways to promote mitochondrial biogenesis, further enhancing ATP production (Eckert et al., 2013) (Table 4).

**TABLE 4:** Potential regulatory roles of polyphenols in the pathogenesis of schizophrenia.

Mechanism	Dose	Model	Target point		Polyphenol
Dopamine	50, 100 mg/kg	Swiss male mice	Dopamine	Ben-Azu et al. (2024a)	Silymarin
	1.25, 2.5, 5, 10 mg/kg	Sprague–Dawley rats	Dopamine, DOPAC	Xu et al. (2005)	Curcumin
	4.80 mM	*Drosophila melanogaster*	Dopamine, SOD, CAT, H_2_O_2_	Filošević Vujnović et al. (2023)	Quercetin
	10, 100 μM	Male and female Wistar Albino rats	Dopamine, DOPAC	Gürsoy and Büyükuysal (2008)	Resveratrol
Glutamic acid	3, 10, 30, 50, 100, 200, 300 μM	SD rats	Glutamic, Ca^2+^, PKC, PKA	Lu et al. (2013)	Quercetin
	25, 50 mg/kg	Swiss experimental mice	GAD	Ben-Azu et al. (2024b)	Naringin
	0.1, 0.5, 1, 10, 100 μmol/L	SD rats Astrocytes	GS, ROS, 20S proteasome, SDH	Song et al. (2020)	Baicalin
	50 mg/kg	NMRI mice	TNF-α, Glutamic, BDNF	Shokouhi et al. (2020)	Silymarin
	0.625, 1.25, 2.5, 5, 10, 20, 40 μM	SD rats	BDNF, TrkB, LDH	Wang et al. (2008)	Curcumin
	1, 10, 25, 50, 100, 250 μM	C6 glioma cells	GS, SDH, S100B, Glutamic acid uptake	dos Santos et al. (2006)	Resveratrol
	1, 5, 10, 20, 50, 100 μM	SD rats	Glutamic, EAAT2, TBARS	Fernandes et al. (2015)	EGCG
BDNF	50, 100 mg/kg	Swiss male mice	MPO, TNF-α, IL-6, BDNF, GSH, CAT, SOD	Ben-Azu et al. (2024a)	Silymarin
	10 , 20 mg/(kg day)	SD rats	BDNF, TrkB, p-Akt, cleaved caspase-3	Yao et al. (2012)	Quercetin
	0.02 mg/kg/day, 0.2 mg/kg/day	Male Kunming mice	BDNF, pTrkB, TrkB, pCREB, CREB, pERK, ERK	Yan et al. (2019)	Kaempferol
	100 mg/kg	C57BL/6J mice	Wnt/β-catenin, GSK3β, BDNF, Akt	Lou et al. (2024)	Curcumin
	40 mg, 80 mg	SD rats	SIRT1/CREB/BDNF, SOD, CAT, Gpx	Niu et al. (2020)	Resveratrol
	2 g/L, 255 mg/kg	C57BL/6J mice	TNFa, IL-1b, IRS/AKT, ERK/CREB/BDNF, NT-3, NT-4, Leptin, Resistin	Mi et al. (2017)	EGCG
	50 mg/kg, 20 mg/kg 100 mg/kg	SD rats	Caspase-3, PI3K/Akt, GSK-3β, CREB/BDNF/Bcl-2	Zuo et al. (2016)	Baicalin
	0, 20, 50, 100 μM	Glial cell, neuron			
Oxidative stress	100 mg/kg	C57BL/6	Keap1/Nrf2/HO-1, MDA, SOD, GSH, CAT, Ach, AChE	Cheng et al. (2024)	Quercetin
	50 µM, 100 µM, 150 µM, 200 µM, 250 µM	BV2 microglia	Nrf-2/HO-1, ROS, NF-κB, IL-6, iNOS, COX-2, HIF-1α, PARP, caspase-3	Kim et al. (2022)	EGCG
	30 mg/kg	SD rats	Keap1-Nrf2-ARE, MDA, NO, CAT, GPx, GR, NQO-1, HO-1, GCLM, p62, CHOP, GRP78	Liao et al. (2023)	Curcumin
	80 mg/kg	Wistar albino rats	DA, DOPAC, HVA, TH, GR, GPx, MMP, Caspase-9, Caspase-3, CI CII CIV CV, RCR	Garabadu and Agrawal (2020)	Naringin
	60 mg/kg	BALB/C	Claudin-5, ZO-1, ROS, MDA, SOD, Nrf2, HO-1, NQO1	Wang et al. (2021)	Baicalin
	4 μg/mL, 8 μg/mL	bEnd.3 cell			
	21 mg/kg	Albino Wistar rats	GSK3β-Nrf2, Ache, GPx, CAT, SOD	Hussein et al. (2018)	Kaempferol
Immune response	10, 30, 50 µM	e BV2 microglia cell	JAK2, STAT3, NO, IL-6, IL-4, IL-10, IL-1β, TNF-α, iNOS, , PI3K/Akt, NF-κB, SOCS-1, M1, M2	Porro et al. (2019)	Curcumin
	50, 100 µg	SJL/J mice,	JAK-STAT, IL-12, T cell, IFNγ, STAT3, STAT4, JAK2, TYK2	Muthian and Bright (2004)	Quercetin
	20 mg/kg, 40 mg/kg	BTBR mice	JAK1-STAT3, IL-6, TNF-α, IFN-γ	Ahmad et al. (2018)	Resveratrol
	5, 10, 20 μM	BV-2 cells	JAK/STAT3, IL-1β, IL-6, TNF-α, IL-10, IGF-1, iNOS, arginase 1	Li et al. (2022)	Naringin
	5, 10, 20, 40 μM	HNSCC cells	JAK/STAT3, Bcl-2, VEGF, Mcl-1, Cyclin D1, IL-6	Lin et al. (2012)	EGCG
Mitochondrial energy metabolism	0.01, 0.05, 0.1, 0.5, 1, 5 μg/mL	C57/BL6 mice	ATP, MMP, OCR, ROS, NADH dehydrogenase	Kang et al. (2020)	Quercetin
	100 mg kg-1 day	C57BL/6 mice	MMP, ATP, ROS	Wang et al. (2015)	Naringin
	0.3 mg/kg	ICR mice	MMP, ATP	Chen et al. (2017)	Resveratrol
	0.1 µM, 1 µM, 5 µM	NT2-N cells	ATP, MMP, OCR, ECAR	Liu et al. (2024b)	Baicalin
	10 µM	Wistar rats	COX, ATP, PGC-1α , p-AMPKα, HO-1/Nrf-2, ROS	Hsieh et al. (2020)	EGCG
	1 mg/kg	SD rats	TCA , NAA, ADP, NAD, Ca^2+^	Chitturi et al. (2019)	Kaempferol
	500 mg/kg	SAMP8, SAMR1	ATP, MMP, ETS	Eckert et al. (2013)	Curcumin

### 7.6 Summary of mechanisms

Plant-derived polyphenolic compounds display therapeutic properties on the brain through various mechanisms, which include boosting antioxidant defenses, reducing neuroinflammation, modulating the balance of neurotransmitters, increasing levels of brain-derived neurotrophic factor (BDNF), and enhancing mitochondrial function. These compounds provide prolonged protective benefits by neutralizing reactive oxygen species (ROS) and activating the Nrf2/ARE pathway, which facilitates the synthesis of endogenous antioxidant enzymes. Moreover, polyphenols influence dopamine signaling pathways, mitigating symptoms associated with heightened dopaminergic activity, such as those seen in schizophrenia. In addition, these compounds promote neuroprotection and enhance cognitive abilities by engaging the BDNF/TrkB signaling pathway to raise BDNF expression. Finally, polyphenolic compounds play a role in achieving better long-term results by encouraging mitochondrial biogenesis and aiding in the restoration of atypical neurodevelopment.

## 8 Clinical research on the improvement of schizophrenia by polyphenols

Currently, clinical trials investigating the effects of polyphenols in schizophrenia remain limited, with most focusing on their use as adjunctive therapies. A controlled clinical trial on curcumin reported promising results, showing its potential efficacy as an adjunct to antipsychotic medications in alleviating negative symptoms. These findings may open new avenues for safe and innovative treatment strategies in the management of schizophrenia (Miodownik et al., 2019). Additionally, baicalin has shown potential benefits in clinical studies as an adjunctive therapy, demonstrating improvements in negative symptoms and cognitive deficits in patients with schizophrenia (Miao et al., 2020). Areoo Samaei et al. observed in clinical trials that the addition of resveratrol to risperidone therapy exhibited beneficial effects in alleviating negative symptoms in patients with chronic schizophrenia (Samaei et al., 2020). A clinical trial is currently underway in China to evaluate the efficacy and safety of quercetin in improving cognitive impairment associated with schizophrenia (Chinese Clinical Trial Registry).

Future research in translational science should emphasize a thorough examination of the mechanisms at play, especially focusing on the regulation of key neurotransmitters like dopamine, serotonin (5-HT), and glutamate. In the context of epigenetic regulation, investigations ought to look into how plant polyphenols affect gene expression, particularly their possible impact on genes related to schizophrenia, which could unveil new therapeutic targets for treatment. Additionally, it is essential to refine drug development processes, which includes innovations in drug delivery methods and improvements in formulations. Moreover, to assess the long-term efficacy and safety of plant polyphenols, there is a need for more extensive clinical trials that incorporate prolonged follow-up studies and research on targeted populations.

## 9 The relationship between multifactor pathological mechanism and schizophrenia

**FIGURE 2:**
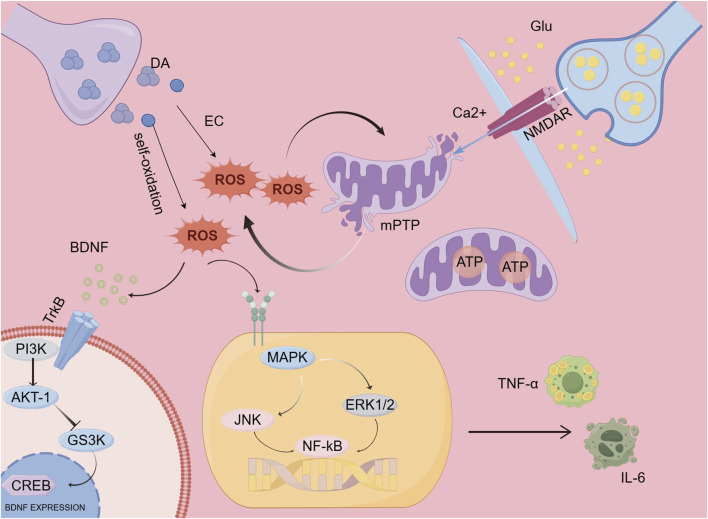
Mechanistic model.

Changes in neurotransmitters are the primary cause of the onset of schizophrenia, particularly alterations in dopamine and glutamate. Dopamine can generate free radicals through multiple pathways, the primary processes involved include enzyme-catalyzed oxidation and auto-oxidation. Auto-oxidation describes the transformation of dopamine molecules into free radicals, a reaction facilitated by redox enzymes, which results in the generation of harmful free radicals. These free radicals can have a negative impact on neurons (Zhou et al., 2023). Increased glutamate release or impaired EAAT function leads to excitotoxicity when glutamate uptake is compromised (Magi et al., 2019; Zhang et al., 2024). Excessive extracellular glutamate persistently activates NMDA receptors, causing sustained Ca^2+^ influx, which overwhelms intracellular calcium homeostasis, damaging mitochondria, and promoting oxidative stress (Parsons and Raymond, 2014; Hardingham and Bading, 2010). The primary cause of oxidative stress stems from an imbalance between reactive oxygen species (ROS) and antioxidants, which results in cellular harm and impairment. This phenomenon is acknowledged as a significant pathological mechanism underlying schizophrenia. Research has indicated that heightened oxidative damage and a diminished ability of the cellular redox regulation system are frequently observed in individuals with schizophrenia, irrespective of any previous treatment with antipsychotic drugs (Goh et al., 2021; Koga et al., 2016). Mitochondrial respiratory chain impairment, changes in membrane permeability and structure, as well as disruption of mitochondrial protective mechanisms, can be triggered by reactive oxygen species (ROS). Nonetheless, the connection between oxidative stress and mitochondrial dysfunction is reciprocal; mitochondrial issues can also lead to the release of ROS, which may worsen oxidative stress (Guo et al., 2013). ROS, as highly reactive oxidants, can reduce BDNF levels through multiple pathways. One possibility is that ROS can directly damage BDNF molecules, impairing their normal function. Another possibility is that ROS activate signaling pathways, such as the Akt-1/GSK3 (glycogen synthase kinase 3) pathway, which is critical for BDNF expression, thereby suppressing its expression and synthesis. Specifically, ROS can activate protein kinases in these pathways, leading to the activation of negative regulatory factors for BDNF gene transcription and thus inhibiting its expression. To summarize, various mechanisms through which ROS affect the expression and function of BDNF could play a role in decreasing BDNF levels (Azimzadeh et al., 2024). Microglia act as the key immune effector cells in the central nervous system, and their activation is closely associated with the onset of schizophrenia, particularly through the release of pro-inflammatory molecules. The production of reactive oxygen species (ROS) can activate the MAPK/NF-κB pathway, which results in the secretion of inflammatory cytokines from microglia, consequently enhancing their activation. Upon activation, microglia generate significant quantities of ROS (Block et al., 2007; Ye et al., 2016). Thus, a vicious cycle forms between microglia and ROS, leading to more severe neuroinflammation and damage (Figure 2).

In conclusion, schizophrenia results from the interplay of multiple pathogenesis mechanisms. In treating such disorders, there is a need to transition from the “single-target” approach to a strategy focused on “multi-target network regulation”. The fundamental concept behind multi-target drug therapy is to concurrently influence various interconnected pathological processes, thereby attaining more comprehensive therapeutic results. For example, research has demonstrated that drugs designed for single targets frequently encounter difficulties in managing the complex networks of diseases in clinical environments, resulting in issues such as drug resistance and side effects. On the other hand, multi-target drug therapy, often referred to as “combination therapy” or “cocktail therapy,” presents an innovative strategy for drug discovery. By focusing on several pathways, it has the potential to more comprehensively affect disease progression while minimizing the likelihood of adverse effects (Yuan et al., 2025). When addressing mental disorders, therapies that focus on a single target can show effectiveness in specific situations, but they often come with unintended adverse effects. This occurs mainly because their narrow target specificity does not consider the patient’s complete biochemical landscape. For instance, when treating depression, commonly used single-target medications often lead to metabolic issues like weight gain or digestive problems. Such side effects can significantly hinder patient compliance with treatment and adversely affect their quality of life (Lee et al., 2025).

## 10 Conclusion

Schizophrenia is a multifaceted neurological condition that encompasses various pathological processes, which include imbalances in neurotransmitters like dopamine and glutamate, as well as oxidative stress, neuroinflammation, and dysfunction of the mitochondria. Additionally, conventional antipsychotic medications, Although they are successful in reducing positive symptoms, there is only minimal enhancement in cognitive deficits, and these treatments frequently come with significant side effects, including metabolic syndrome. This study systematically investigates the mechanisms by which various plant-derived polyphenols exert therapeutic effects on the core pathological processes of schizophrenia through multi-target actions. Furthermore, based on nanotechnology and metabolic regulation, we propose an innovative delivery strategy for polyphenols. This approach provides a novel perspective for the development of high-efficiency, low-toxicity natural derivatives, offering promising potential for schizophrenia treatment.

Traditional drug development has often focused on single targets (e.g., dopamine D2 receptors); however, the treatment of schizophrenia requires a more systemic intervention strategy. This study reveals that the multi-dimensional mechanisms of polyphenols can overcome the limitations of single-target therapies. For instance, quercetin inhibits voltage-dependent calcium channels *via* the PKC/PKA signaling pathway, reducing glutamate release in the synaptic cleft. Similarly, silymarin downregulates dopamine levels in the striatum, alleviating hyperkinetic symptoms. Curcumin stands out for its ability to regulate the dopaminergic system in a bidirectional manner, as it can reverse dopamine depletion found in the prefrontal cortex, while also mitigating excessive dopamine activity in the striatum. Additionally, the approach of addressing both oxidative stress and inflammation is especially significant. The capabilities of polyphenols as antioxidants go beyond simple ROS scavenging; they also trigger the Nrf2/ARE pathway, which promotes the production of endogenous antioxidant enzymes, thereby forming a lasting protective barrier. Furthermore, resveratrol acts to inhibit the JAK1-STAT3 pathway, preventing microglial cells from polarizing toward the M1 phenotype, and decreasing the secretion of pro-inflammatory factors such as IL-6 and TNF-α. This combined blockade effectively interrupts the detrimental cycle linking oxidative stress and neuroinflammation, showcasing enhanced neuroprotective effects in schizophrenia models in comparison to inhibitors that target only one mechanism. Beyond the aforementioned mechanisms, polyphenols can also improve the long-term prognosis of schizophrenia by enhancing mitochondrial biogenesis and reconstructing neurodevelopmental abnormalities. For example, EGCG activates the SIRT1 pathway, promoting mitochondrial function and respiratory chain complex activity, restoring ATP production efficiency. EGCG treatment enhances mitochondrial membrane potential and significantly improves ATP turnover rates. Additionally, polyphenols can activate the BDNF/TrkB pathway; for instance, curcumin upregulates BDNF expression in the hippocampus *via* the Wnt/β-catenin signaling pathway, reversing schizophrenia-like phenotypes.

The important pharmacological effects of polyphenols are notable; however, their clinical application has been seriously constrained by their low solubility in water, extensive metabolism during the first pass, and restricted ability to cross the blood-brain barrier. However, recent studies have shown that functionalized nanocarriers, such as nanoparticles, liposomes, and polymeric microparticles, can effectively encapsulate quercetin and control its release rate, thereby improving bioavailability. A metabolic symbiosis strategy involves the co-administration of resveratrol with specific probiotics, which can enhance bioavailability. This “gut-brain axis” delivery approach may pave a new avenue for the precise regulation of natural products.

The experimental data of polyphenols demonstrate their relatively good efficacy; however, the clinical application of polyphenol-based therapies still faces numerous challenges. Certain polyphenols, at high concentrations, may exert pro-oxidative effects, potentially leading to cellular damage. This suggests the importance of understanding the dose-effect relationship and establishing personalized dosing windows. Dose optimization algorithms based on pharmacokinetic models may help balance efficacy and safety. Gender differences also play a role, as seen with silibinin, which significantly increases BDNF levels in male mice but shows no such effect in female individuals, likely due to the cross-regulation of estrogen receptors. Future studies on polyphenols should incorporate gender-stratified analyses to avoid therapeutic biases. Additionally, the development of real-time monitoring technologies is necessary to mitigate potential risks.

Future research should focus on innovative approaches to bioavailability and delivery technologies, developing novel nanodelivery systems to enhance the solubility, blood-brain barrier permeability, and targeting capabilities of polyphenols. Investigating chemical modifications, such as glycosylation, on the stability and pharmacokinetic properties of polyphenols is essential. Exploring the interactions between gut microbiota and polyphenol metabolism could lead to strategies using probiotics to enhance bioavailability. Carrying out multicenter, double-blind, randomized controlled clinical trials is essential for assessing the effectiveness of polyphenols, whether used alone or as complementary therapies, in enhancing both positive and negative symptoms, along with cognitive abilities, in individuals with schizophrenia. Creating patient stratification models that incorporate biomarkers, including plasma BDNF levels and markers of oxidative stress, will facilitate accurate predictions regarding dosage and treatment oversight. Assessing the long-term safety of polyphenol treatment, with particular attention to hepatic and renal function, metabolic parameters, and potential drug interactions, is necessary. Investigating the combination of polyphenols with conventional antipsychotic medications may reveal their potential to mitigate metabolic side effects. Investigation into how polyphenols influence synaptic plasticity during crucial neurodevelopmental phases, like adolescence, may enhance understanding of their use in early interventions. Additionally, examining the therapeutic possibilities of polyphenols in comorbid conditions, including depression and metabolic syndrome linked to schizophrenia, requires more in-depth research.

In summary, polyphenols of plant origin have shown tremendous potential in the treatment of schizophrenia. Polyphenolic compounds have the potential to alleviate symptoms of schizophrenia and enhance cognitive function through various mechanisms. These include boosting antioxidant protection, diminishing neuroinflammation, balancing neurotransmitters, improving responses to oxidative stress and immune challenges, elevating levels of brain-derived neurotrophic factor (BDNF), and promoting mitochondrial activity. Despite the significant progress made in current research, several issues remain to be further explored. For instance, the bioavailability and pharmacokinetic properties of polyphenols need further optimization to improve their effective concentrations and duration of action in the body. Moreover, data from extensive clinical trials remain inadequate, necessitating further research to confirm the effectiveness and safety of polyphenols across various patient demographics. Subsequent studies ought to concentrate on the molecular pathways influenced by polyphenols, examining their combined effects with alternative therapeutic strategies, as well as creating new polyphenol derivatives and delivery mechanisms to improve treatment results and patient adherence. In summary, polyphenols sourced from plants offer fresh perspectives and techniques for addressing schizophrenia and may serve as a valuable complementary strategy for upcoming treatments targeting this disorder.
